# Video Sequence Segmentation Based on K-Means in Air-Gap Data Transmission for a Cluttered Environment

**DOI:** 10.3390/s23020665

**Published:** 2023-01-06

**Authors:** Przemyslaw Mazurek, Dawid Bak

**Affiliations:** 1 Department of Signal Processing and Multimedia Engineering, West Pomeranian University of Technology in Szczecin, 70-310 Szczecin, Poland; 2 IBM Polska Sp. z o.o., 02-255 Warszawa, Poland

**Keywords:** air gap, image segmentation, k-means, security, digital modulation

## Abstract

An air gap is a technique that increases the security of information systems. The use of unconventional communication channels allows for obtaining communication that is of interest to the attacker as well as to cybersecurity engineers. One of the very dangerous forms of attack is the use of computer screen brightness modulation, which is not visible to the user but can be observed from a distance by the attacker. Once infected, the computer can transmit data over long distances. Even in the absence of direct screen visibility, transmission can be realized by analyzing the modulated reflection of the monitor’s afterglow. The paper presents a new method for the automatic segmentation of video sequences to retrieve the transmitted data that does not have the drawbacks of the heretofore known method of growth (filling) based on an analysis of adjacent pixels. A fast camera operating at 380 fps was used for image acquisition. The method uses the characteristics of the amplitude spectrum for individual pixels, which is specific to the light sources in the room, and clustering with the k-means algorithm to group pixels into larger areas. Then, using the averaging of values for individual areas, it is possible to recover the 2-PAM (pulse-amplitude modulation) signal even at a 1000 times greater level of interference in the area to the transmitted signal, as shown in the experiments. The method does not require high-quality lenses.

## 1. Introduction

The security of IT systems is extremely important due to various types of threats. Due to the increase in the complexity of information systems, the number of potential attack methods has increased. Additionally, these attacks can be launched from anywhere in the network, which makes them easier to carry out and increases the chance of an attack. The security of IT systems is usually based on the use of classic security systems such as firewalls, anti-viruses (AVs), intrusion detection systems (IDSs), or security information and event management (SIEM), but in the case of critical infrastructure, they prove to be insufficient, and the best possible solution seems to be complete isolation from the mains. This solution has a number of disadvantages as it hinders data exchange, which is sometimes needed. This type of insulation is known as an air gap. This type of isolation can vary in scale, ranging from single computers to large networks.

The use of an air gap is efficient from a security point of view, but does not mean that this type of network cannot be hacked. The most obvious is to infect the air gap network directly and then gain control over it remotely (breaking the air gap) [1]. This means that there is a need to obtain a communication channel, simplex or duplex, which will provide an opportunity to obtain valuable data or take control of the infrastructure. Of course, such attack operations are known to those skilled in the cybersecurity field, and various passive or active means are used to prevent and detect such attacks.

An example of passive measures is the disconnection of communication interfaces (especially wireless ones) that are most vulnerable to attacks. An active measure is, for example, monitoring the activity of devices connected to the network in order to detect anomalies.

There are many methods that allow for air gap transmission. As standard methods of wired and wireless communication are unavailable, there are also many [1] non-standard one-way (simplex) transmission channels: acoustic, magnetic, visible optical, and thermal.

As these channels are usually unidirectional, they are used for the transmission of sensitive data; in particular, for the transmission of passwords in order to, for example, open a wireless communication channel. The question of how a computer is infected is beyond the scope of this work.

**Figure 1:**
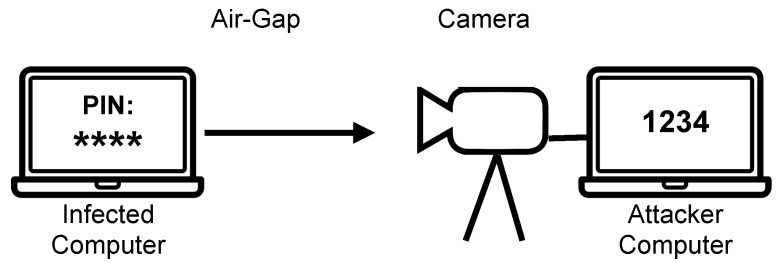
Scheme of the attack using air gap video transmission.

One very interesting method is data transmission using the brightness modulation of a computer screen. The screen may or may not be directly visible (by reflection from the walls and equipment of the room). The use of common methods of protection against screen viewing, such as tarnishing glass, does not reduce the usefulness of this method. Moreover, this method allows for transmission over a very long distance because the use of a telescope with a large aperture and long focal length allows for the observation of screen modulation. It was shown in [2] that this method does not require any special permissions and can be implemented, for example, in Linux from the user level, because changing the display brightness is available to the user. Additionally, it is possible to implement this method in various ways: by changing the screen content or by changing the screen backlight (Figure 1).

Brightness modulation cannot be strong because it can be seen by a human, especially a computer operator. However, it is possible to modulate the brightness in a way that is unnoticeable to a human but in a way where it is still possible to estimate the degree using a high-quality camera and image processing algorithms.

### 1.1. Related Works

Communication with the use of a computer screen or TV set allows for performing two tasks.

The first is intentional communication with, for example, the user’s smartphone. Using a dedicated smartphone application that supports the camera, the user can point the camera at the screen so that, if possible, the entire monitor image is visible, sending additional information without using any other type of network. One of the goals of the researchers is to increase the transmission speed.

The second task is air gap transmission, where there is no requirement for the direct visibility of the entire computer screen. All methods supporting the transmission of this type, which should not be detected, are interesting for the attacker; on the other hand, knowledge about the possible forms of attack may allow for the implementation of a new tool to protect computers and detect attack attempts. Speeds in this type of attack are dependent on the modulation method and are fractions of bits per second when using frequency-shift keying (FSK) modulation and up to several bits with pulse-amplitude modulation (PAM) [3].

The known techniques of air gap transmission are based on the encoding and modulation of the signal in the light of the [4] LEDs; for example, those found in network devices [5,6], signaling the operation of the hard disk [7], or signaling the keyboard status [8,9]. Similar techniques can be used to transmit data from mobile devices [10] via LEDs in these devices [11] or to transmit data from surveillance cameras via infrared [12]. Methods based on the use of a computer screen [13] and a camera as a receiver [14,15] are an extension of the above concepts. Methods were developed using LCD screens as transmission transmitters [16] in which various signal modulation and coding techniques were used [17,18,19,20,21,22], as well as techniques for decoding such a transmission in real time [23]. Previous studies dealt with the possibility of transmitting a signal through the LCD screen by an appropriate modulation of the frequency of screen brightness changes [13] and changes in the screen backlight brightness [2]. The next study focused on the possibility of improving the transmission efficiency by using recorded image segmentation and analyzing only designated areas [24]. Based on the analysis of previous works by various teams, it seems that the next step towards improving the presented ones should be to enable the effective receiving of the transmitted data in the absence of the direct visibility of the screen from which the signal is transmitted.

### 1.2. Content and Contribution of the Paper

**Figure 2:**
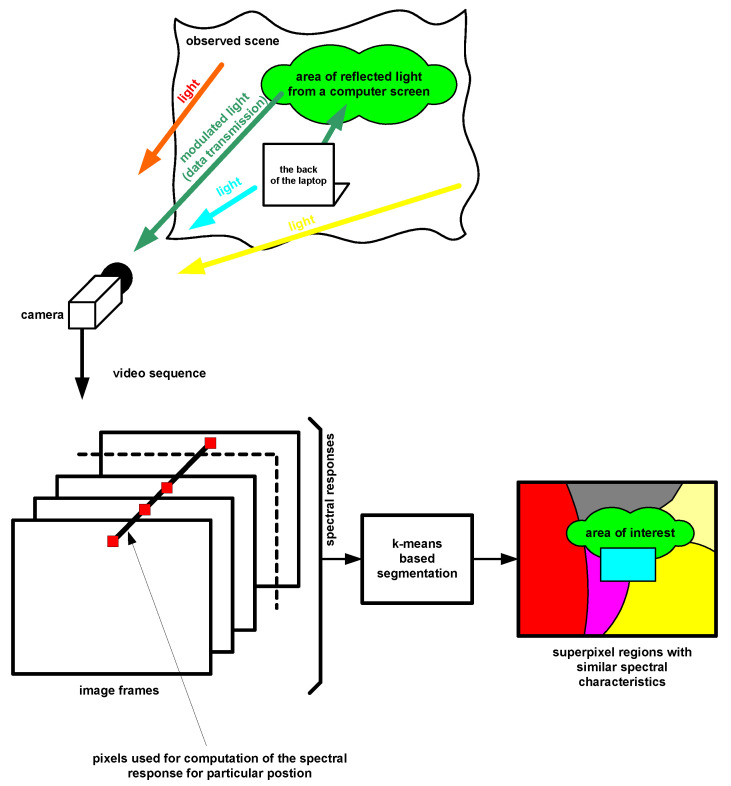
Scheme of the attack using air–gap video transmission using the proposed method.

The paper proposes and verifies an automatic approach to segmentation of video sequences in order to detect image regions in which air-gap video transmission takes place (Figure 2).

Air gap transmission using the modulated light of a computer screen is difficult to receive in the case of many light sources, for example in a room. In extreme cases, the light from the monitor is not available directly, but only as a reflection from the wall or objects. This means that the recorded video sequence contains a mixture of multiple light sources. Image segmentation based on the frequency characteristics of fast-changing brightness waveforms of light sources allows to determine regions with similar characteristics and, in the next step, to detect transmissions in them. By specifying the regions, it is possible to improve the SNR (Signal-to-Noise Ratio) to reduce the transmission error rate.

The main contributions of this paper are:

- A proposal of the use of the amplitude spectrum for changes in pixel values in the time domain for video sequences for data pre-processing;
- A proposal of the use of the Spatio-Temporal Segmentation for pixel group detection;
- A proposal of the use of the k-means algorithm [25] for unsupervised clustering of the amplitude spectrum associated with pixels or pixel groups;
- An experimental demonstration that shows that the method allows for effective clustering in conditions of strong interference, where the reflected signal from the wall is approximately 1000 times weaker than the interference (another monitor);
- A demonstration that the method is suitable for high-speed data transmission using 2-PAM in relation to the slow FSK method.

The method of air gap communication with the use of brightness modulation and acquisition is presented in Section 2. In Section 2 and Section 3, two methods for signal estimation are presented, including a proposed algorithm for the automatic segmentation of video sequences. In order to segment the acquired image, we propose the use of the k-means algorithm for signal spectrum obtained from video sequence. This spectrum is calculated for particular pixel position in video sequence.

The results for the two communication scenarios are presented in Section 3 and Section 4. A discussion of the proposed method and the results obtained is presented in Section 4. Final conclusions and further work are provided in Section 4 and Section 5.

This paper shows the possibility of recovering the correct, but noisy and distorted, 2-PAM signal. The use of an equalizer and the demodulation process, including the recovery of the clock signal, are beyond the scope of this paper.

## 2. Materials and Methods

### 2.1. Data Transmission Using Screen Brightness Modulation

**Figure 3:**
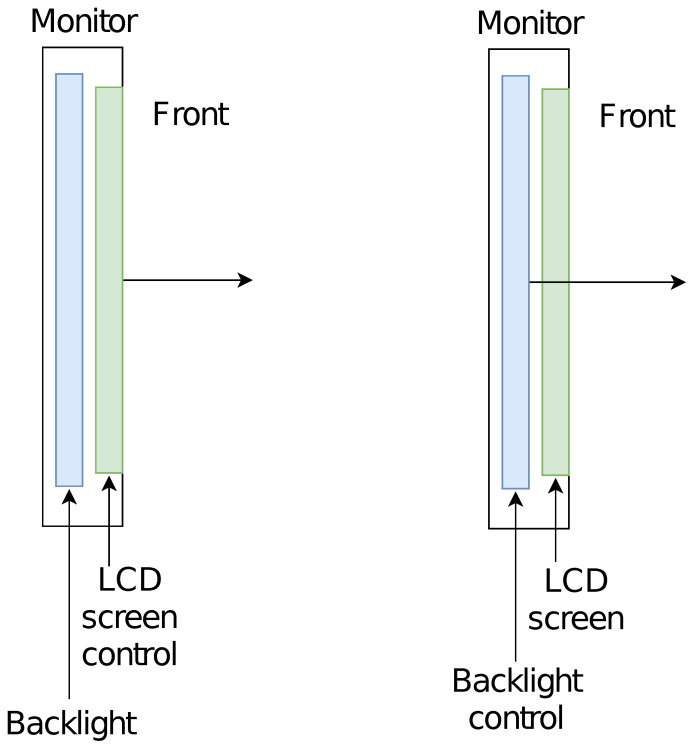
Construction of the LCD monitor (cross-section on the side): transmission method with the use of LCD matrix modulation transmittance modulation (**left**) and using backlight modulation (**right**).

There are two methods of modulating the brightness of the screen. The first is based on the modulation of the transmittance of the LCD screen. It can be implemented in two ways: by changing the pixel values of the image or by changing the settings related to the brightness and contrast of the controller. The control scheme is shown in Figure 3 (left).

The second method uses screen backlight brightness modulation and can be used for compact fluorescent lamp (CFL) backlight (older monitor models) and LED backlight (currently used). The control scheme is shown in Figure 3 (right). Both types of backlight use pulse-width modulation (PWM)-controlled converters. The value of the fill factor affects the brightness of the backlight and thus the monitor screen.

Brightness modulation in both cases can use different methods. The simplest is FSK, which requires a relatively large number of frames allocated to a single transmitted bit. Its advantage is a high resistance to interference. The main disadvantage is a very slow transmission; for example, 0.1 bps. In the case of PAM modulation, two brightness levels of 2-PAM can be used, with a small number of image frames allocated to a single bit; therefore, the transmission rate can be even several bits per second. Such a transmission is more susceptible to various disturbances, but it allows us to send much more information, which is important for the attacker.

### 2.2. Video Image Acquisition Equipment

The ASI290MM-COOL USB 3.0 camera (manufactured by ZWO) was used in the image acquisition experiments. This camera includes a Peltier module for matrix cooling (not used) and is connected via USB 3.0 to a computer. The matrix sensor (Sony IMX290) is a CMOS type with a rolling shutter. Acquisition software (ASICAP v.1.6.2) enables the adjustment of acquisition parameters; in particular, the region of interest (ROI). Region of interest (ROI) reduction increases the number of recorded frames per second at the expense of image resolution. In the experiments, 380 fps were set, which allows for image acquisition along with artifacts resulting from screen refresh and matrix backlight. The image resolution is 320×240 pixels with the Binning=2 setting. This camera captures a monochrome image using the 8-bit-per-pixel mode. The use of a monochrome sensor without an IR filter allows it to achieve a very high sensitivity in relation to typical color cameras with a Bayer filter matrix and an IR filter.

A Canon EFS 18–55 mm lens was used, with the focal length set to 55 mm. It is a cheap kit lens and sufficient for air gap transmission applications. The distance between the camera and the computer was approximately 9 m. By using cheap long-focal-length lenses (for example, telescopes), even greater distances can be obtained. The quality of the lens—for example, the correction of chromatic aberration, distortion, and coma—is not important. In the experiments, the focus was not even precisely adjusted, so the image from the camera is blurry. However, it is important to use a tripod to correctly record the video sequence.

**Figure 4:**
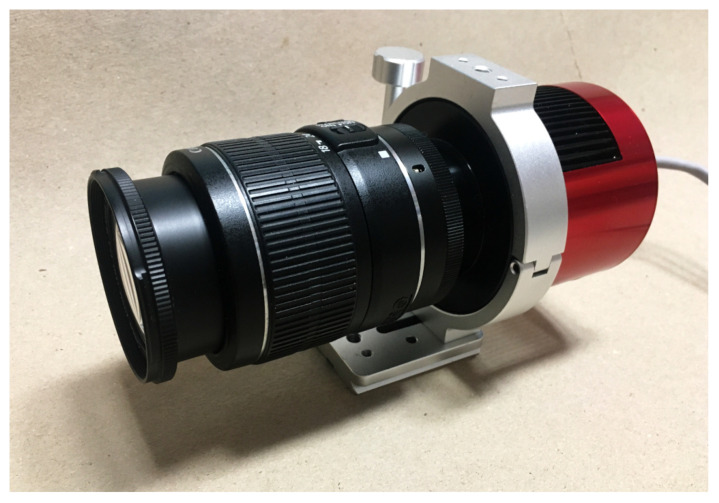
Camera with lens used in an experiment.

The camera, lens, camera lens adapter, and tripod mount are shown in Figure 4.

### 2.3. Observation Scenarios

**Figure 5:**
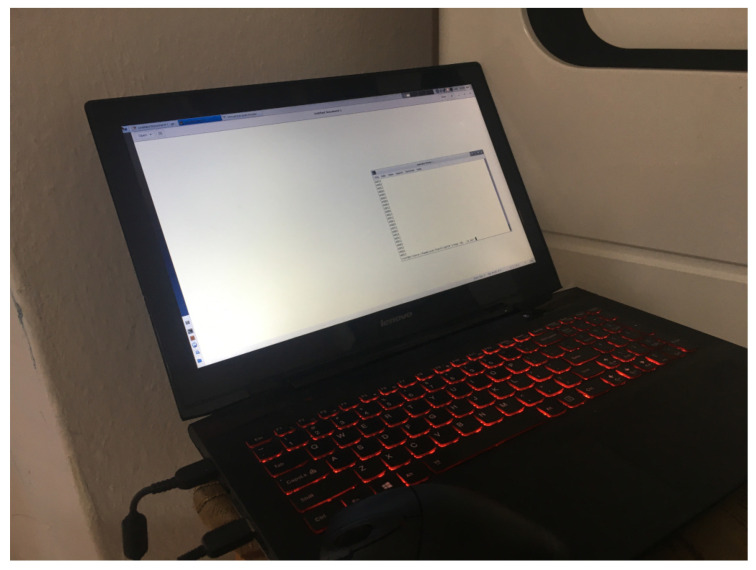
Scene view with the computer screen directly visible to the camera.

**Figure 6:**
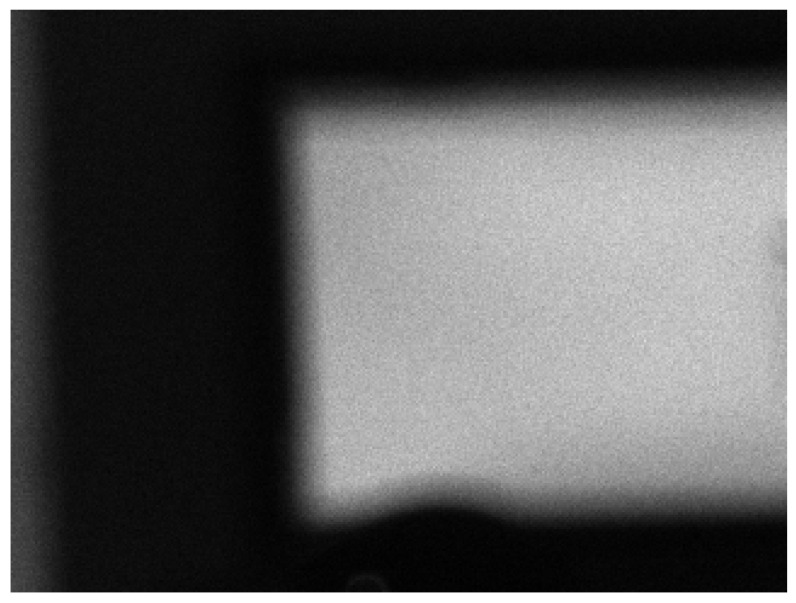
Sample image frame from the recorded sequence (resolution 320×240 with intentional lack of focus).

Two sample scenarios were tested. The first is based on the direct visibility of the transmitting computer screen (Figure 1). It does not necessarily have to be a high-quality video sequence. The image may be blurred due to the lack of sharpness or the use of a special matting foil on the glass of the room to protect the monitors from viewing the screen content. An exemplary image of a computer with installed software transmitting by modulating the brightness of the backlight is shown in Figure 5. A sample image frame from the recorded sequence (resolution 320×240 with an intentional lack of focus) is shown in Figure 6.

**Figure 7:**
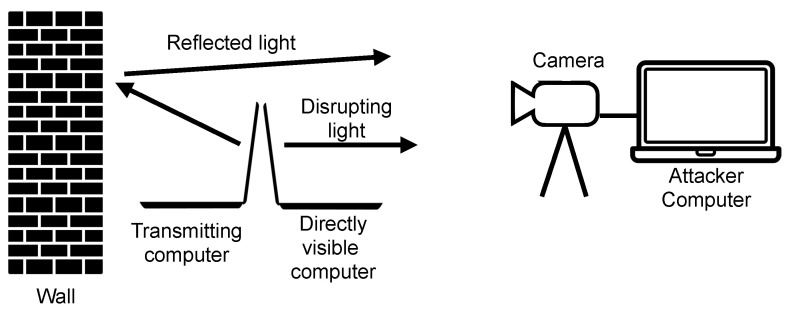
Diagram of a transmission experiment in cluttered environment.

**Figure 8:**
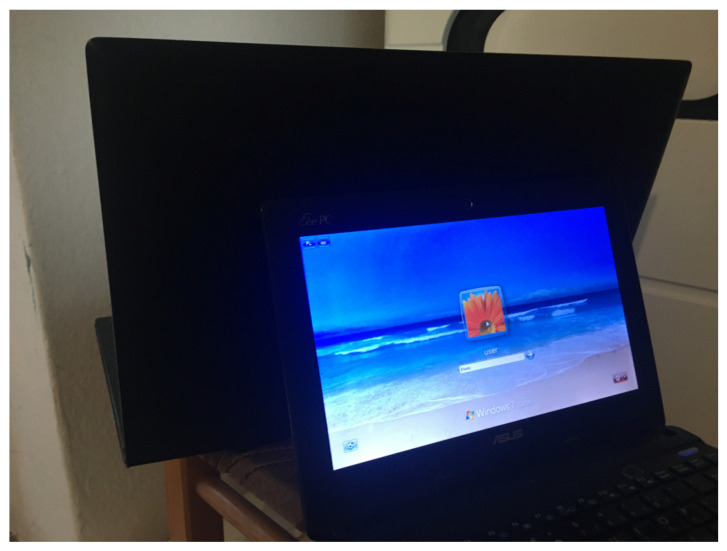
Scene view with two computers. A computer with a screen directly visible to the camera is a source of interference. The transmission is made by the computer screen facing away from the camera by reflection from the wall.

**Figure 9:**
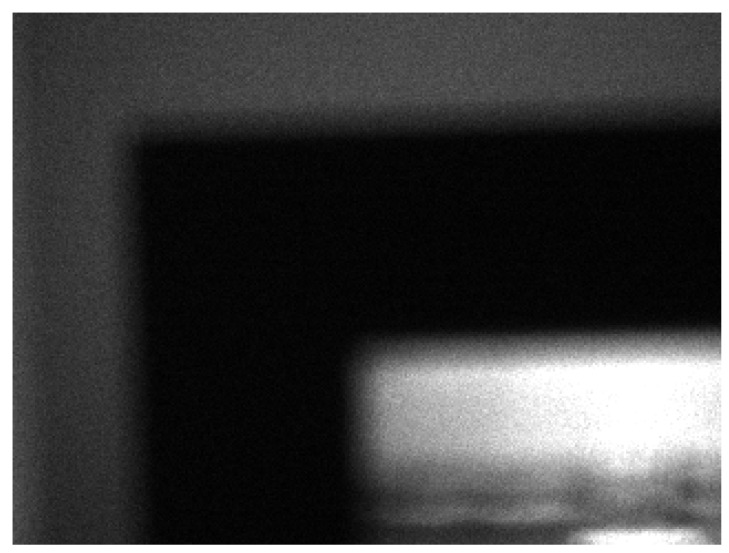
Sample image frame from the recorded sequence (resolution 320×240 with intentional lack of focus).

The second test scenario concerns the transmission visibility only through the reflection of light from the wall (Figure 7). The screen of the transmitting computer is not visible to the camera. Additionally, a second computer was set up with the screen directly disible to the cameras. This means that there is a strong source of jamming signals for the camera. Two cases were investigated for this test scenario: the application of the basic algorithm to change the average brightness of the whole image frame and the application of the proposed segmentation algorithm to selectively average the transmitted signal. An exemplary image of a computer with installed software transmitting by modulating the brightness of the backlight is shown in Figure 8. A sample image frame from the recorded sequence (resolution 320×240 with an intentional lack of focus) is shown in Figure 9.

### 2.4. Simple Data Communication Channel and the Estimation of the Transmitted Signal

The communication channel can be of two types. The first type is the simplest and assumes that the image frame is converted to a single pixel. In this case, image averaging is used according to the formula:

(1) $$M\left(n\right)=\frac{1}{x_{N}\cdot y_{N}}\sum_{x=1}^{x_{N}}\sum_{y=1}^{y_{N}}X\left(x,y\right)$$

where M(n) is the average image value for the *n*-th image frame, *x* and *y* are the image pixel coordinates of the recorded *X*, and the *X* image has a resolution of $x_{N}\times y_{N}$. This type of approach is very simple and assumes that the acquisition conditions are very good. This case is the reference scenario.

### 2.5. Interference in the Communication Channel

In more realistic cases, there are several sources of interference: camera sensor noise (which can be reduced by cooling the matrix), screen activity that changes the brightness of the image, and room lighting of various types of lamps—incandescent, fluorescent, discharge, or LED—controlled by various direct-current-to-direct-current (DC–DC) or alternating-current-to-direct-current (AC–DC) converters. The screens also do not glow uniformly because they are controlled by DC–DC converters. Other computer screens are also a serious source of interference. The orientation of the transmitting computer screen may also be disadvantageous; for example, it may be turned away from the camera. Despite such an unfavorable orientation, it is possible to observe the transmission by the reflection from the environment (walls, objects, and even stationary people).

Data reception in the case of many sources interfering with transmission requires filtration. In the event of interference from lighting lamps, it is possible to use bandpass filters for the 100 Hz or 120 Hz band for the 50 Hz and 60 Hz mains, respectively.

This means that the received signal M(n) consists of components that must be separated from each other. Furthermore, the air gap transmission signal is very weak, and in the case of working with a reflection, it is additionally weakened. Unfortunately, this is a very difficult task in many cases. It is possible to use a known training sequence to further separate the signal, but it is not a good approach. The fundamental error is the use of an operation represented by the formula (1), which causes the loss of spatial information about the signal.

### 2.6. Multi-Dimensional Data Communication Channel

The second type of communication channel description is more complex but significantly reduces the impact of interference.

The transmitted air gap signal is not a one-dimensional signal, but a multi-dimensional signal in the sense of a transmission channel, since it may cover all or part of the image pixels. The air gap transmission is a transmission with $x_{N}\times y_{N}$ independent transmission channels; for example, for a resolution of 320×240, there are 76,800 channels. Of course, there is a greater or lesser correlation between individual channels (pixels), but individual channels (pixels) are affected to varying degrees by noise. Since the signal is weak, it is necessary to apply formula (1) not for the whole image, but for the best area captured by the camera. This means that image segmentation must be performed. Blind signal separation methods are possible, but the task is still complex. It is possible to use the usual methods of image segmentation—for example, to define an area of the walls—but this is not very efficient because it is not guaranteed that a given area contains the reflected signal. Moreover, large areas—for example, a wall—do not necessarily reflect the signal completely, but only in a small area. Unnecessarily increasing the analysis area causes the inclusion of other interfering sources in the spatially localized signal.

It is necessary to perform segmentation, not based on the context of the scene, but using the characteristics of the signal itself on the video sequence. This is the key task for the receiving system.

**Figure 10:**
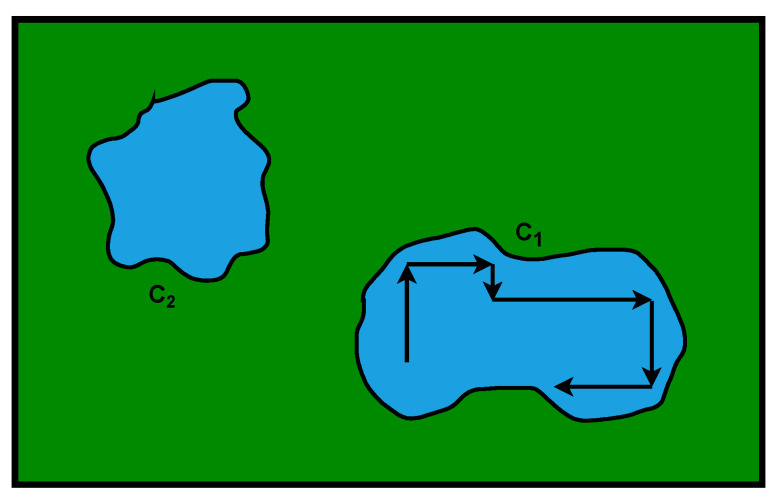
Scheme of the camera image with the problem of segmentation using neighborhood analysis algorithms.

There is a large spatial correlation between the transmitted signals for individual pixels. The work [24] proposes an approach using a kind of growth algorithm (filling an area based on the similarity of neighboring pixels). Unfortunately, this method is quite limited as it works like a fill algorithm and maintains its limitations. Assuming two separate spaces (Figure 10), the areas where there is transmission $C_{1}$ and $C_{2}$, it is possible to locate the area $C_{2}$ but not the connection with the area $C_{1}$. In the case of a wrong starting point (strong interference), the wrong area may be marked.

Due to the problem of the methods from the work [24] and the need to segment the image with the maximization of the averaged area of where the transmission is, even if they are separate areas, a more effective method was proposed.

### 2.7. Proposed Method

**Figure 11:**
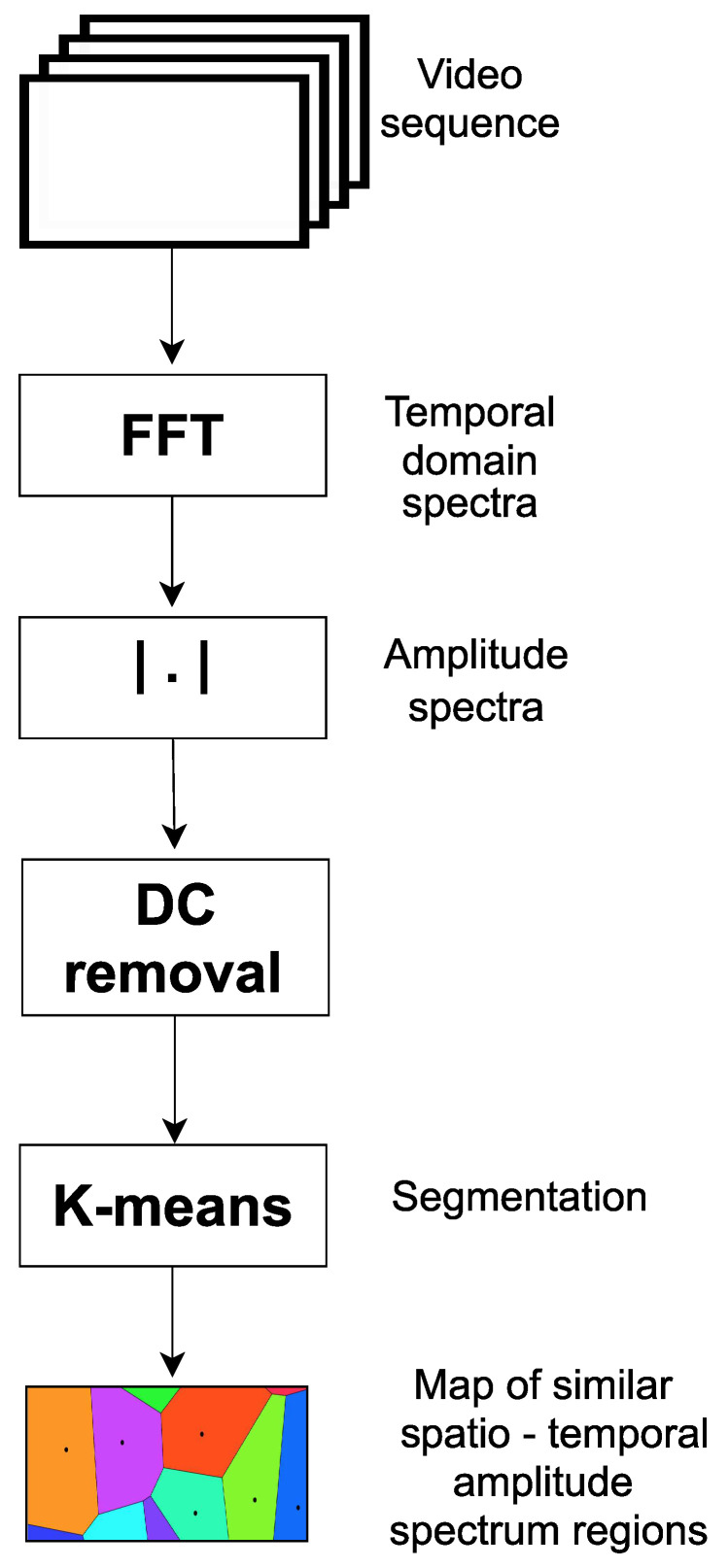
Diagram of algorithm.

The proposed segmentation method uses spatio-temporal clustering to recover the transmitted information (Figure 11).

#### 2.7.1. Video Sequence

Each pixel of the image recorded by the camera contains information about the light sources in the environment, both the lamps and the backlight of the monitors. Some light sources can be continuous light sources (DC component only), which is possible for certain types of lamps and natural lighting. Most of the lighting comes from artificial sources that are controlled by frequency or by changing the duty cycle (PWM). This means that most light sources have their own specific frequency characteristics, related to their quick switching on and off in order to obtain a certain brightness. In the case of lamps, pulsed operation of light sources is usually used, which are powered in such a way that electricity is supplied in short periods of time and in other periods it is not supplied. This allows to simplify the power supply systems for light sources, which are often low-voltage receivers (e.g., LED) and the power supply is high-voltage (e.g., 110 or 230 V). In the case of computer screens, it is related to the screen refresh rate and backlight brightness adjustment.

Depending on the location of the light sources and the environment, we obtain an image of a scene in which individual pixels are the result of mixing signals, which are very fast time changes in the brightness of pixels from light sources. In the case of some light sources, the frequency of their flashing is of the order of several tens of kHz, because then the converter systems often generate sounds (coils or transformer) but inaudible to humans.

Using a very fast camera, it is possible to directly measure these changes for each pixel, according to the sampling theorem. The brightness value for each pixel then contains the component sum of all variable brightness of the light sources.

It is also possible to use fast cameras that do not meet the requirements of the sampling theorem, which results in aliasing. Despite the violation of the sampling theorem, it is possible to use these types of signals, because the goal is not to recover a fast-changing signal from every light source, but controlled slow-changing transmission signals.

Since each pixel X(x,y) contains a mixture of signals from all (*V*) light sources $L_{v}\left(x,y\right)$, it is necessary to group pixels into groups with similar frequency characteristics. Otherwise, more light sources are mixed and the transmission quality deteriorates.

The signal value for the particular pixel is:

(2) $$X\left(x,y,t\right)=\sum_{v=1}^{V}L_{v}\left(x,y,t\right),$$

where $L_{v}$ is the brightness of the *v*-th light source at time *t*, and *x* and *y* are the pixel coordinates in the image.

In the extreme case, only one pixel may contain data transmission, but due to the level of noise in the image sensor and the light sources themselves, it leads to a deterioration of the SNR (Signal-to-Noise Ratio). The second extreme case is when the entire image is treated as a source of data transmission. In this case, an attempt is made to recover the transmission from the overall brightness of the image recorded by the camera. This is not the right solution because it may contain much more pixels not related to the real transmission and reflections of other light sources, which also worsens the SNR.

In order to solve the SNR problem, we proposed an adaptive approach, where the algorithm tries to find the optimal region of the image, giving a high SNR. The basis for this is the spatial relationships between pixels. There is a high chance that neighboring pixels have similar signals. This process uses signal clustering using the k-means algorithm.

This allows for automatic determination of regions with similar characteristics, but only some of them are interesting and contain transmission data.

The number of all regions is *K*. This means that the entire *X* image (strictly a video sequence) is divided into *K* areas:

(3) $$X=\sum_{k=1}^{K}C_{k},$$

where $C_{k}$ is the particular region/class/superpixel.

**Figure 12:**
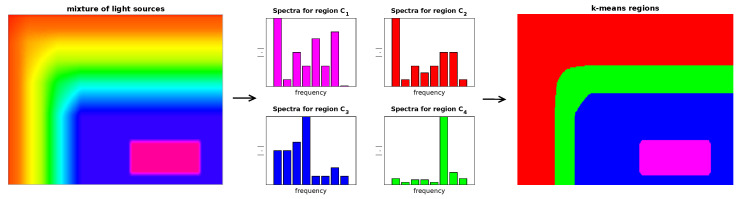
Scheme of the segmentation video sequence using spectral analysis using similarity of the amplitude spectrum for the time domain (false colors were used for better visualization for classes $C_{1},C_{2},C_{3},C_{4}$).

While this division in work [24] results from the growth algorithm, the proposed method (based on k-means) is more flexible: two distant areas can be considered as one, allowing SNR to be improved based on signal similarity. The proposed similarity of signals uses frequency similarity, which allows to create k regions of similarity. Figure 12 shows an example for k = 4. The signal similarity corresponds to the segmentation of the video sequence.

#### 2.7.2. Temporal Domain and Amplitude Spectra

Individual pixels of the image contain the sum of signals from all $L_{v}$ sources. Using the Discrete Fourier Transform - DFT (preferably Fast Fourier Transform - FFT) we determine the frequency spectrum $W_{f}\left(x,y\right)$ for each pixel position (exactly all pixel values in the video sequence t=0,⋯,(T−1)):

(4) $$W_{f}\left(x,y\right)=\sum_{t=0}^{T-1}X\left(x,y,t\right)\cdot e^{-\frac{j\cdot 2\cdot \pi }{T}f\cdot t},$$

where $W_{f}$ is the complex value of the spectrum for the *f*-th band of the spectrum, *T* is the number of frames of the video sequence and *t* is the frame number. Individual frequency bands are f=0,⋯,T−1.

We consider the amplitude spectrum $|W_{f}|$ as a data points of k-means algorithm.

#### 2.7.3. DC Suppression

The amplitude spectrum beyond the DC component was used. This is due to the fact that the constant component is related to the average brightness, which does not transmit any information. Typically this is the brightness of the wall or the average brightness of the screen. Since the reflection of the light with the transmitted signal may concern, for example, a wall of different brightness due to a specific texture, it is necessary to treat these areas as one, and suppression the DC signal allows this.

This operation consists in zeroing the first component (DC, f=0) of the spectrum:

(5) $$|W_{0}\left(.,.\right)|=0.$$

#### 2.7.4. Clustering Using k-Means Algorithm

**Figure 13:**
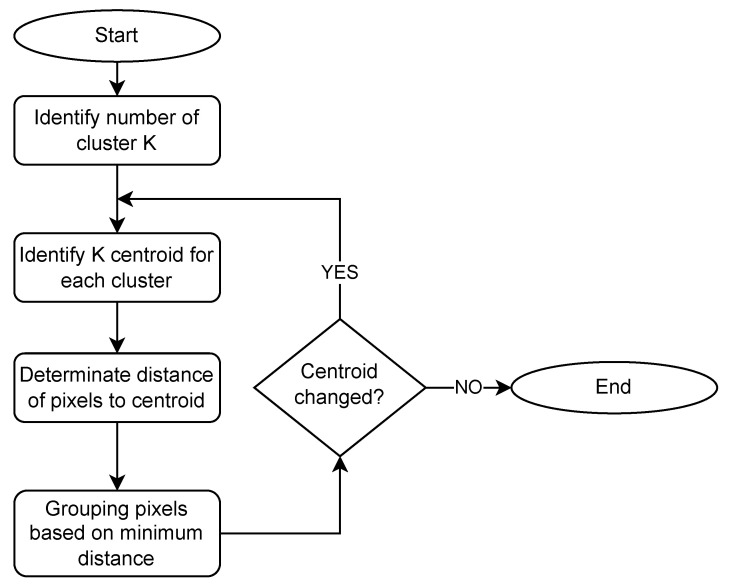
k-means algorithm.

K–means clustering is one of the most commonly used clustering algorithms. k-means algorithm allows grouping of pixels with similar spectrum for estimation region creating superpixels shown in Figure 2 and Figure 12. Basics steps of k-means algorithm used to image segmentation is shown in Figure 13 and described below:

1. Choose the number of clusters you want to find which is k,
2. Randomly assign the data points $|W_{f}|$ to any of the k clusters,
3. Then calculate the center of the clusters,
4. Calculate the distance of the data points $|W_{f}|$ from the centers of each of the clusters,
5. Depending on the distance of each data point from the cluster, reassign the data points to the nearest clusters,
6. Again calculate the new cluster center,
7. Repeat steps 4, 5 and 6 till data points don’t change the clusters.

The k-means algorithm groups all spectra into $C_{k}$ classes (k=1,⋯,K), which allows to obtain a final image with a division shown in colors (Figure 12) and further testing the presence of data transmission in individual regions.

#### 2.7.5. Setting Parameters and Optimization of Computations

Depending on the angle of view of the camera, the resolution and the size of the objects in the scene, image scaling can be applied to reduce the computational cost.

This article proposes the use of the k-means algorithm with the Euclidean distance function. To reduce the amount of computation, the input image (320×240) was scaled to a ten times smaller resolution (32×24). Scaling depends on the size of the observed computer in the video image. The scaling factor should be chosen based on the original size of the visible screen. For example, if, on an original video sequence, we a had 10 × 10-pixel screen, we can downscale it a maximum of 10 times to 1 pixel, but not more than that.

A total of 1024 consecutive image frames were used for the spectral analysis. This is a relatively small number, but such a small set shows that the operation can be performed very quickly. Since the sampling rate is 380 Hz, less than 3 s of data transmission recording is sufficient. Since 2-PAM data transmission uses a speed of 20 bps, relatively many bits are transferred during this time period. Increasing the number of frames allows for segmentation with a greater accuracy.

Each pixel position in the original or reduced image corresponds to a sequence of 1024 values for the same position. The use of 1024 frames allows us to take advantage of the speed advantage of the fast Fourier transform (FFT) algorithm, which is used for each sequence separately. For the scaled-down image (32×24), it is 768 FFT operations. The transformation results in a 3D data block of size 32×24×(1024−1).

The obtained 3D block is converted to a matrix of 768×1024 because typical implementations of the k-means algorithm operate on matrices, not volumes (3D). The k-means algorithm performs clustering for a given number of expected clusters. The selection of the number of clusters depends on the scene and it is possible to automatically select the number of clusters. The k-means algorithm provides individual cluster indexes that are assigned to individual image pixels.

The next step is to average the pixel values for each image frame according to the obtained segmentation regions. Although the segmentation is based on 1024 frames, it is possible to use the segmentation obtained for further frames if the scene does not change.

## 3. Results

### 3.1. Direct Observation of Computer Screen

**Figure 14:**
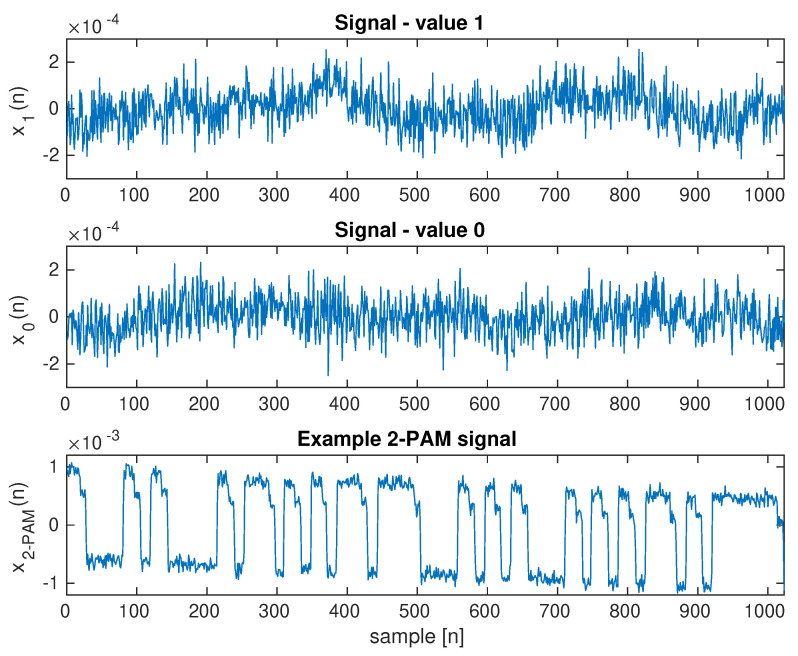
Waveforms for the average brightness of video sequence frames. Waveforms for two brightness levels and a random transmitted sequence with 2–PAM modulation are shown. Brightness modulation with LCD backlight.

**Figure 15:**
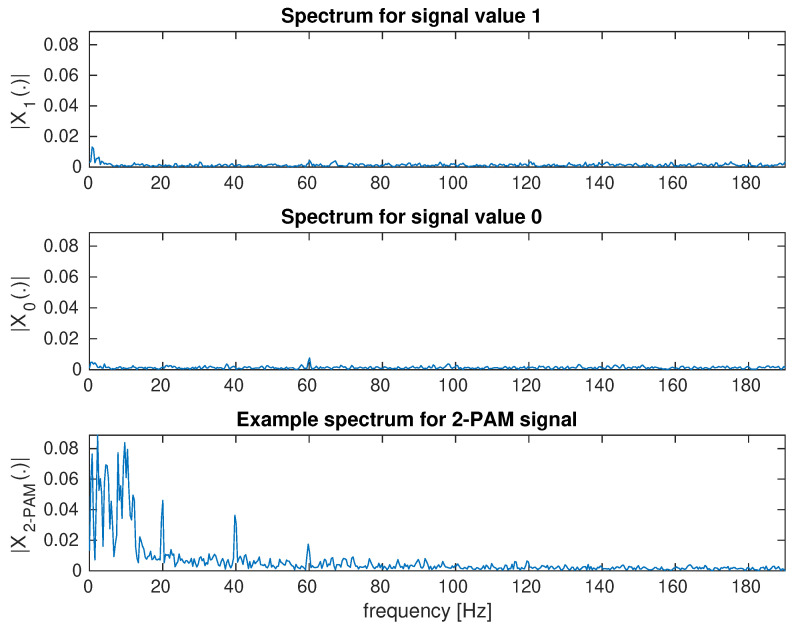
Signal spectra for the average brightness of the frames of the video sequence. Spectra for two brightness levels and a random transmitted sequence with 2–PAM modulation are shown. Brightness modulation with LCD backlight.

Figure 6 shows an example of a frame of a video sequence recorded with a camera. The screen area is large in relation to the entire frame and, due to direct visibility, it is possible to correctly estimate the transmitted signal (Figure 14). Additionally, two signals for continuous transmitting, 0 and 1, are shown (Figure 14), which show a noise floor that is five times lower than the transmitted signal. This case is shown for the purpose of a comparison to a very complex scenario. Figure 15 shows the spectrum of signals: continuous 0, continuous 1, and a random bit sequence with the 2–PAM modulation.

### 3.2. Indirect Observation (Reflection from the Wall) of Computer Screen

**Figure 16:**
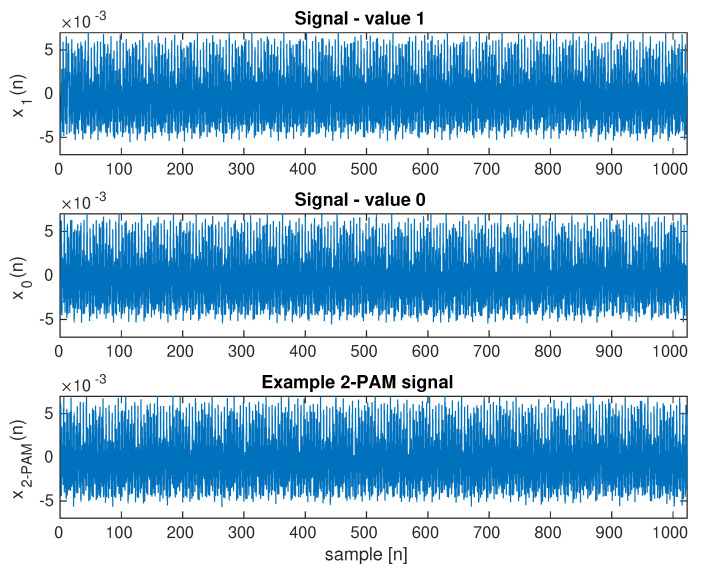
Waveforms for the average brightness of video sequence frames. Waveforms for two brightness levels and a random transmitted sequence with 2-PAM modulation are shown. Brightness modulation with LCD backlight. Scenario with strong brightness disturbances.

**Figure 17:**
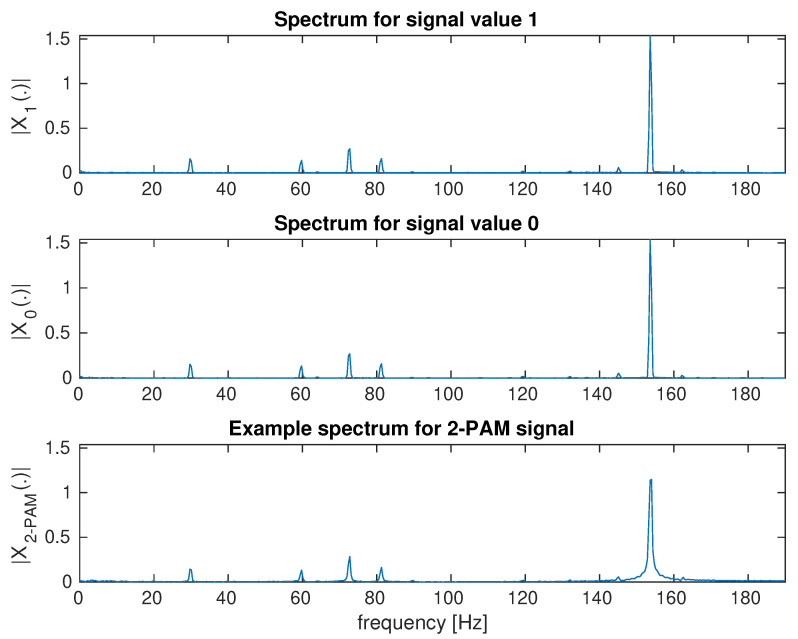
Signal spectra for the average brightness of the frames of the video sequence. Spectra for two brightness levels and a random transmitted sequence with 2-PAM modulation are shown. Brightness modulation with LCD backlight. Scenario with strong brightness disturbances.

Figure 9 shows an exemplary frame of an image from a video sequence for a scenario of light reflection from the screen of a monitor transmitting data. The second computer’s screen, which is directly visible, is a source of interference. In the case of using the averaging of the whole image frame, the 2-PAM signal is completely lost (Figure 16). In the waveforms of continuous signals 0 and 1, the disturbance from this monitor is visible. Figure 17 shows the amplitude spectra of the signal, in which the dominant component is the interference from this monitor.

### 3.3. Indirect Observation (Reflection from the Wall) of Computer Screen—Using the Proposed Method

**Figure 18:**
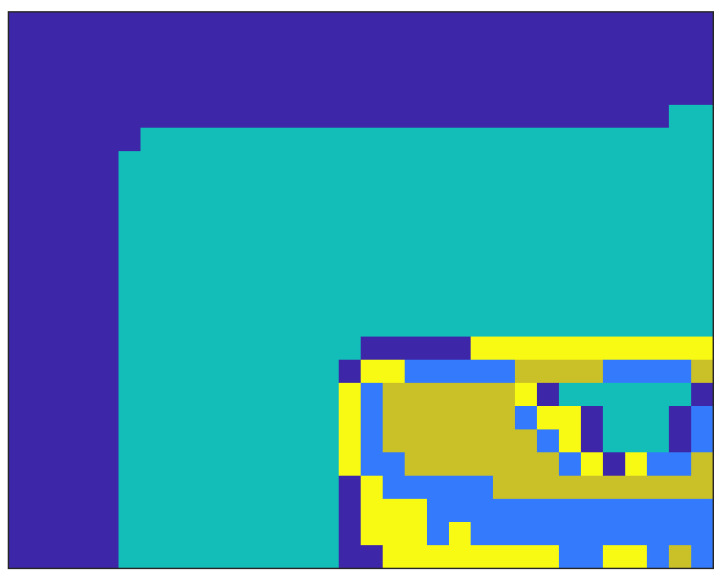
Image segmentation obtained with the k-means algorithm for the amplitude frequency spectrum in the time domain.

The use of the proposed segmentation method allows for a reduction in the averaging area. The segmentation result for the number of clusters k=5 is shown in Figure 18. The 2-PAM transmission area is the wall region marked in dark blue in Figure 18.

**Figure 19:**
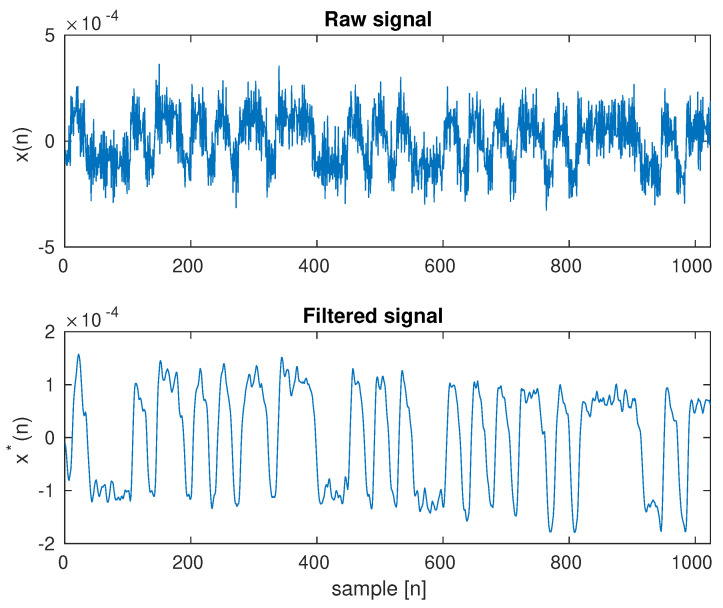
Waveforms for the selective brightness of video sequence frames. Raw and filtered waveforms a random transmitted sequence with 2-PAM modulation are shown. Brightness modulation with LCD backlight. Scenario with strong brightness disturbances.

Figure 19 shows the raw waveform of the 2-PAM signal for the selected area and the waveform after filtering the signal using a Butterworth low-pass filter of order 2 and a cut-off frequency of 50 Hz.

## 4. Discussion

The results of the experimental research show the advantages of using the proposed method. Averaging the whole image is a simple method that allows for recovering the 2-PAM signal. The signal peak value is approximately 0.0018 (Figure 14), with most of the image frame being occupied by the monitor (Figure 6). This value may depend on the brightness of the screen and the modulation depth (value difference between the level for 0 and 1 signal). Increasing the modulation depth would improve the SNR value, but a limiting value for the modulation depth would be selected for this monitor so that it is not yet visible. Further increasing the modulation depth makes it visible. The quality of the received 2-PAM signal is very good (Figure 14), and the signal itself has not been subjected to a filtering process—in particular, equalization—which is typically used in digital communication channels on the receiving side. The 2-PAM signal has steep slopes due to the lack of low-pass filtering. The demodulation process, including the recovery of the clock signal (frequency and phase), is relatively simple.

The second scenario with the image reflected from the wall shows that the basic method of averaging the whole image is not suitable. With the direct visibility of the screen, the spectrum components are maximum 0.09 (Figure 15). For the second scenario, the signal level is over 1. This means that the interfering signal is over 100 times stronger. Since the useful signal is reflected from the wall and visible only in a small area, the noise level is much greater than 100 times. The averaging attempt leads to useless results because the transmitted 2-PAM signal is lost in disturbances (Figure 16).

The interfering signal has several frequency components shown in Figure 17. An attempt was made to digitally filter the signal to separate the transmitted and interfering signals, but this is very difficult because some of the interfering components are in the lower frequency range characteristic of the transmitted signal.

The only option is to define areas where there is a specific spectrum of signals and look for such an area that makes it possible to recover the transmitted signal, which was proposed in [24]. The use of the amplitude spectrum according to the proposed method, after clustering with the k-means algorithm, allowed us to determine statistically similar areas (Figure 18). The main area (wall) was properly delimited. It can be compared with Figure 8, where a characteristic glow from the screen is visible on the wall.

The number of clusters is an open problem. Too small a number causes an aggregation of areas with different frequency characteristics (different disturbances). Too high a number leads to a weakening of the SNR since the basis of a high SNR is averaging using more pixels. This is an open research problem that is very much dependent on the scene, which may be unique.

The study used k=5 clusters, and the k-means algorithm correctly marked areas on the wall, but also on the monitor of the interfering computer. It is possible to use morphological algorithms to reduce the number of small areas that may be unrelated spatially. However, these areas are the result of an unsupervised classification scheme, so it may not be right to reject them in some cases. There was no relationship between them in the experiment. In general, the correct selection of the value of k is a theoretical issue. It is possible to optimize the parameter for individual data transfer by checking successive k values and checking the correctness of data reading. In this case, a checksum for the transmitted data should be used. Of course, the use of a checksum is necessary in the practical implementation of the discussed method because, in the absence of data transmission, it is necessary to suppress the chance of incorrect data reading. The results in Figure 19 show the signal received, despite the lack of additional morphological filtering or the use of a priori information. The 2-PAM signal is approximately eight times weaker than in the direct visibility experiment. This is a result of the losses from the signal reflection from the wall because the monitor surface Figure 6 is similar to the wall surface (Figure 9). The signal reflected from the wall was approximately 1000 times weaker than the interfering one. This shows the scale of the problem and, at the same time, the advantages of the proposed solution.

In addition to the presented method, it is also possible to use semantic segmentation, which is currently a very active area of research [26,27,28,29,30]. There are many works on similar topics where semantic algorithms have been used to determine the areas. Thanks to this approach to the problem, it is possible to quickly determine the areas where the signal is reflected. However, in many cases, the use of such methods will not bring the expected effect; for example, when the wall is illuminated, the semantic segmentation will select one large area and the data reading will be incorrect. The use of semantic segmentation can be helpful in determining important areas; however, it cannot be the basic method. A similar effect can also be obtained by background estimation methods [31], where the use of background estimation techniques seems to be a good method for improving the efficiency of the detection of transmission areas in the proposed method [32]. The support of the presented method, with the help of semantic segmentation and background estimation, is probably a good starting point for further work in the discussed topic.

## 5. Conclusions

The advantage of the proposed solution is very fast area estimation. In the case of an attacker, it is extremely important, because the lack of a proper signal may indicate the need to quickly change the position in relation to, for example, the room with the transmitting computer. The method also has a very low computational cost, as the FFT implementations are very efficient and this operation is only performed once at the beginning of the observation. Further operations are related to simple averaging/accumulation, which is very effective with modern processors with single instruction, multiple data (SIMD) architecture.

The proposed method works effectively, as shown in the experiment, for a difficult case. The case of multiple monitors bouncing off the same wall, where there is a mixture of transmitted and interfering signals, can be more complex. This is a topic that will be considered in the next work.

## Data Availability

Not applicable.
